# Overexpression of Efflux Pumps, Mutations in the Pumps’ Regulators, Chromosomal Mutations, and AAC(6′)-Ib-cr Are Associated With Fluoroquinolone Resistance in Diverse Sequence Types of Neonatal Septicaemic *Acinetobacter baumannii*: A 7-Year Single Center Study

**DOI:** 10.3389/fmicb.2021.602724

**Published:** 2021-03-11

**Authors:** Subhasree Roy, Somdatta Chatterjee, Amrita Bhattacharjee, Pinaki Chattopadhyay, Bijan Saha, Shanta Dutta, Sulagna Basu

**Affiliations:** ^1^Division of Bacteriology, Indian Council of Medical Research (ICMR)-National Institute of Cholera and Enteric Diseases, Kolkata, India; ^2^Department of Neonatology, Institute of Post-Graduate Medical Education and Research, Kolkata, India

**Keywords:** *A. baumannii*, neonatal sepsis, sequence types, ciprofloxacin/levofloxacin/moxifloxacin, RND pumps, AdeRS/AdeL/AdeN, *gyrA*/*parC*, India

## Abstract

This study investigates susceptibility toward three fluoroquinolones (ciprofloxacin, levofloxacin, moxifloxacin), multiple fluoroquinolone-resistance mechanisms, and epidemiological relationship of neonatal septicaemic *Acinetobacter baumannii*. Previous studies on fluoroquinolone resistance in *A. baumannii* focused primarily on ciprofloxacin susceptibility and assessed a particular mechanism of resistance; a more holistic approach was taken here. Epidemiological relationship was evaluated by Multi Locus Sequence Typing. Minimum Inhibitory Concentrations of fluoroquinolones was determined with and without efflux pump inhibitors. Overexpression of efflux pumps, resistance-nodulation-cell-division (RND)-type, and multidrug and toxic compound extrusion (MATE)-type efflux pumps were evaluated by reverse transcriptase-qPCR. Mutations within regulatory proteins (AdeRS, AdeN, and AdeL) of RND-pumps were examined. Chromosomal mutations, presence of *qnr* and *aac(6*′*)-Ib-cr* were investigated. *A. baumannii* were highly diverse as 24 sequence-types with seven novel STs (ST-1440/ST-1441/ST-1481/ST-1482/ST-1483/ST-1484/ST-1486) were identified among 47 *A. baumannii.* High resistance to ciprofloxacin (96%), levofloxacin (92%), and particularly moxifloxacin (90%) was observed, with multiple mechanisms being active. Resistance to 4^th^ generation fluoroquinolone (moxifloxacin) in neonatal isolates is worrisome. Mutations within GyrA (S83L) and ParC (S80L) were detected in more than 90% of fluoroquinolone-resistant *A. baumannii* (FQRAB) spread across 10 different clonal complexes (CC1/CC2/CC10/CC25/CC32/CC126/CC149/CC216/CC218/CC513). Efflux-based FQ resistance was found in 65% of FQRAB with ≥2 different active pumps in 38% of strains. Overexpression of *adeB* was highest (2.2−34-folds) followed by *adeJ*, *adeG*, and *abeM*. Amino acid changes in the regulators (AdeRS/AdeN/AdeL) either as single or multiple substitutions substantiated the overexpression of the pumps. Diverse mutations within AdeRS were detected among different CCs whereas mutations within AdeN linked to CC10 and CC32. Chromosomal mutations and active efflux pumps were detected simultaneously among 64% of FQRAB. Presence of *aac(6′)-Ib-cr* was also high (74% of FQRAB) but *qnrS* were absent. As most FQRABs had chromosomal mutations, this was considered predominant, however, isolates where pumps were also active had higher MIC values, establishing the critical role of the efflux pumps. The high variability of FQ susceptibility among FQRAB, possessing the same set of mutations in *gyrA*, *parC*, and efflux pump regulators, was also noted. This reveals the complexity of interpreting the interplay of multiple resistance mechanisms in *A. baumannii*.

## Introduction

*Acinetobacter baumannii* remains in the forefront as a nosocomial pathogen, causing infections and outbreaks in adults and neonates (Qu et al., 2016; Hujer et al., 2017; Gramatniece et al., 2019). Studies from our laboratory have shown the clinical significance of *A. baumannii* infection and colonization among neonates (Roy et al., 2010; Chatterjee et al., 2016). The ability to survive under unfavorable conditions and the propensity to acquire resistance determinants has made infections with this pathogen difficult to treat in intensive care units (Asif et al., 2018).

In comparison to broad-spectrum cephalosporins and aminoglycosides, fluoroquinolones (FQs) are more active in reduction of infections caused by a wide range of Gram-positive and Gram-negative pathogenic bacteria including *A. baumannii.* However, a high rate of resistance to FQs was also detected (Lopes and Amyes, 2013; Ardebili et al., 2015). WHO indicated these antibiotics as the highest priority agents among the Critically Important Antimicrobials for Human Medicine (World Health Organization, 2019).

There are now four generations of quinolone/fluoroquinolone antibiotics in clinical use, among which, the most commonly prescribed FQs in current medical practice are ciprofloxacin, levofloxacin, and moxifloxacin (Redgrave et al., 2014). All FQs target DNA gyrase and topoisomerase IV, involved in the process of DNA replication, with varying efficiency in different bacteria. However, subsequent studies found that in a given bacterial species, different fluoroquinolones have been shown to have different primary targets. The issue of quinolone targeting is still a matter of debate, and the relative contributions of gyrase vs. topoisomerase IV to quinolone action need to be evaluated on a species-by-species and drug-by-drug basis (Ferrara, 2007; Aldred et al., 2014). Chromosomal mutations in the quinolone resistance determining regions (QRDRs) of DNA gyrase subunit A (*gyrA*) or topoisomerase IV subunit C (*parC*) is a well-recognized mechanism of FQ resistance in *A. baumannii* (Redgrave et al., 2014). Another important mechanism is overexpression of efflux pumps (Redgrave et al., 2014). To date, three RND-family (resistance nodulation division) pumps AdeABC, AdeIJK, AdeFGH, and one MATE-family (multidrug and toxic compound extrusion) pump AbeM have been reported to be associated with efflux of FQs in *A. baumannii* (Marchand et al., 2004; Su et al., 2005; Damier-Piolle et al., 2008; Coyne et al., 2010). Efflux pump genes are chromosomally encoded and controlled by regulators. AdeRS, a two-component regulatory system regulates the expression of AdeABC pump. Expression level of AdeFGH is controlled by a LysR-type transcription regulator AdeL whereas AdeN, a TetR-like transcription regulator, represses expression of AdeIJK. In addition, plasmid-mediated quinolone resistance determinants (PMQRs) such as *qnr* have been identified in *A. baumannii*, though infrequently (Touati et al., 2008; Jiang et al., 2014; Yang et al., 2015). Another PMQR, *aac(6′)-Ib-cr* is a variant of an aminoglycoside acetyltransferase that contains two specific point mutations, Trp102Arg and Asp179Tyr. This enzyme modifies only ciprofloxacin and norfloxacin by N-acetylation at the amino nitrogen on its piperazinyl substituent. These two mutations are required for quinolone acetylating activity. Acetylation of fluoroquinolones by AAC(6′)-Ib-cr decrease drug activity and provides low-level resistance to fluoroquinolones (Aldred et al., 2014; Rodríguez-Martínez et al., 2016).

The rate of antimicrobial resistance in India is high. The consumption of FQs is higher in India in comparison to cephalosporins and macrolides (Laxminarayan and Chaudhury, 2016; Farooqui et al., 2018). Empirical treatment for neonatal sepsis, recommended in current WHO guidelines is intravenous ampicillin (or penicillin) plus gentamicin for 7 days. Fluoroquinolones could be an option as second line for sepsis or severe infection due to MDR bacteria. Though the use of this antibiotic is restricted in the pediatric population due to its potential toxicity, judicial and appropriate use of this class of drug can be a choice for the treatment of sepsis among neonates (Fuchs et al., 2016). A thorough evaluation of the susceptibility of these pathogens toward different classes of FQs and the resistance mechanisms would thus make this study clinically relevant.

To date, majority of the studies on fluoroquinolone resistance in *A. baumannii* focused on only ciprofloxacin resistance and studied either chromosomal mutations (Spence and Towner, 2003; Hujer et al., 2009; Park et al., 2011; Liu et al., 2012; Ardebili et al., 2015) or AdeABC pump (Park et al., 2011; Lopes and Amyes, 2013; Ardebili et al., 2014). It is necessary to study the susceptibility of higher generation FQs such as moxifloxacin among diverse sequence types of *A. baumannii* in comparison to all known clinically important older fluoroquinolones over time and correlate the contribution of all possible mechanisms simultaneously in a single isolate. Till now, none of the previous studies have delineated all these mechanisms simultaneously. To fill this gap in knowledge, this study aims to evaluate (i) susceptibility pattern of the three most clinically important FQs (ciprofloxacin, levofloxacin, and moxifloxacin) in neonatal septicaemic *A. baumannii*, (ii) prevalence of different sequence types (STs) among neonatal septicaemic *A. baumannii* in a single center, (iii) role of the chromosomal mutations (*gyrA* and *parC* genes) in FQ resistance, (iv) role of RND and MATE-family efflux pumps in FQ resistance, (v) association of mutations found within regulators of RND pumps with overexpression of efflux pumps, and (vi) role of PMQRs in FQ resistance.

## Materials and Methods

### Bacterial Isolates and Multi Locus Sequence Typing (MLST)

A total of 47 *A. baumannii* were collected by standard procedure from blood of septicaemic neonates admitted to the NICU (Neonatal Intensive Care Unit) of IPGMER hospital, Kolkata, India during 2009–2015. Identification of *A. baumannii* isolates was initially confirmed by the VITEK 2 compact system (BioMérieux, Marcy l’Etoile, France).

Identification of *A. baumannii* was further confirmed by Multi Locus Sequence Typing using the MLST Pasteur Scheme^1^ which uses internal fragments of the seven housekeeping genes (*cnp60*, *fusA*, *gltA*, *pyrG*, *recA*, *rplB*, and *rpoB*). Global optimal eBURST (goeBURST) analysis was performed using the software goeBURST 1.2.1 version available on the website to assign the STs into respective clonal complexes (goeburst.phyloviz.net) and were defined as single locus (SLVs), double loci variants (DLVs), and triple loci variants (TLVs).

### Determination of Susceptibility to FQs

MIC (Minimum Inhibitory Concentration) of FQs was determined using broth microdilution method and resistance was interpreted according to EUCAST breakpoints (European Committee on Antimicrobial Susceptibility Testing) for *Acinetobacter* spp. (ciprofloxacin: resistant > 1 mg/L and levofloxacin: resistant > 1 mg/L) (Rules, 2018). The breakpoint of moxifloxacin was not available for *Acinetobacter* spp. in EUCAST. As breakpoints of resistance for ciprofloxacin and levofloxacin in Enterobacteriaceae (ciprofloxacin: resistant > 0.5 mg/L and levofloxacin: resistant > 1 mg/L) were quite comparable to *Acinetobacter* in EUCAST, thus the moxifloxacin resistance breakpoint for Enterobacteriaceae (resistant > 0.25 mg/L) in EUCAST was used for *A. baumannii*. The MIC_90_ values over time for all FQs were also calculated.

Susceptibility towards other antimicrobials like ceftazidime (resistant ≥ 32 mg/L), cefepime (resistant ≥ 32 mg/L), meropenem (resistant ≥ 8 mg/L), imipenem (resistant ≥ 8 mg/L), amikacin (resistant > 64 mg/L), and minocycline (resistant ≥ 16 mg/L) was determined by VITEK 2 compact system and interpreted according to CLSI (Clinical and Laboratory Standards Institute) breakpoints (Clinical and Laboratory Standards Institute [CLSI], 2019). MIC of colistin was determined by broth microdilution method and interpreted according to CLSI breakpoints (resistant ≥ 4 mg/L).

### Genotypic Characterization of the FQ-Resistant Strains

The QRDRs of the *gyrA* and *parC* genes were PCR amplified and subsequently sequenced (Valentine et al., 2008; Hujer et al., 2009). *A. baumannii* ATCC 17978 was used as reference for sequence comparison. The 5′- and 3′-nucleotide positions of the primers used to amplify the full QRDR region of *gyrA* and *parC*, the annealing temperatures and respective product sizes are mentioned in Supplementary Table S1. RND-family efflux pump genes (*adeB, adeJ, adeG*) and MATE-family efflux pump gene (*abeM*) were screened by PCR and PMQR determinants (*qnrA*, *qnrB*, and *qnrS*) were also checked (Cattoir et al., 2007; Rumbo et al., 2013). *aac(6*′*)-Ib* was amplified with primers known to amplify all known *aac(6*′*)-Ib* variants (Park et al., 2006). Specific bands were digested with BtscI (New England Biolabs, MA) to identify *aac(6*′*)-Ib-cr* responsible for ciprofloxacin resistance (Park et al., 2006).

### Responsiveness of the Strains to Efflux Pump Inhibitors

To understand the extent of involvement of efflux pumps in the FQ resistance phenotype, susceptibility to FQs in the presence of two efflux pump inhibitors (EPI), namely phenylalanine arginine β-naphthylamide (PAβN; Sigma) and 1-(1-naphtylmethyl)-piperazine (NMP; Sigma) at 50 mg/L was tested (Pannek et al., 2006; Blanchard et al., 2014). A significant inhibition was defined as a 4-fold or greater reduction of MIC in the presence of efflux pump inhibitors (Pannek et al., 2006). Previous studies of *A. baumannii* also showed the use of PAβN and NMP at 50 mg/L or even higher (100 mg/L) concentration to establish efflux mediated resistance (Pannek et al., 2006; Valentine et al., 2008; Blanchard et al., 2014). To rule out any bactericidal activity of the inhibitors (PAβN and NMP) at the concentrations used in the study (50 mg/L), MIC value for the two inhibitors was determined in the absence of antibiotics for half of the isolates chosen randomly amongst those showing reduction of FQ MIC in presence of inhibitors.

### Reverse Transcriptase Quantitative PCR (RT-qPCR)

Strains which showed ≥ 4-fold reduction of MIC for FQs were considered for overexpression study of RND-family pump genes (*adeB*, *adeJ*, *adeG*) and MATE-family pump gene (*abeM*) by RT-qPCR. The expression of pump genes was normalized to the housekeeping gene (16S rRNA).

Total RNA was initially extracted from 1 ml (about 10^8^ cells/ml) of mid-logarithmic bacterial cultures using Nucleospin RNA isolation kit (Nucleospin, Macherey-Nagel, Düren). Contaminating DNA was removed by RNase-free DNase I (New England Biolabs, United States). The concentrations and purity of RNA were quantified with a spectrophotometer at 260 and 280 nm (260/280 ratio of > 1.8). Reverse transcription was performed with 2 μg of RNA according to manufacturer’s instructions using the high-capacity cDNA reverse transcription kit (Applied Biosystems, Warrington, United Kingdom).

Quantification of the expression of the target genes was performed with Power SYBR Green PCR Master Mix (Applied Biosystems) using the StepOne Plus Real-Time PCR System and software (Applied Biosystems, United States) according to the manufacturer’s instructions. Oligonucleotide primer sequences used for pump genes and 16S rRNA is shown in Supplementary Table S1. After a 10 min activation of the modified Taq polymerase at 95°C, 40 cycles of 30 s at 95°C, 45 s at 55°C or 58°C and 1 min at 60°C were performed. Data were acquired at 60°C. The relative gene expression (Δ*CT*) for pump genes transcripts was calculated against that for the 16S rRNA gene (Δ*CT* of test gene = *CT* of test gene- *CT* of 16S), and the △△*CT* was calculated against that for the ciprofloxacin-susceptible strain, *A. baumannii* ATCC 19606 (expression = 1), which served as the control. Relative expression level of pump genes was calculated by the 2^–^^ΔΔ^ *^*CT*^* method. An effect on gene expression was considered significant when the corresponding ratios were > 2.0.

### Nucleotide Sequencing of Pump Regulatory Elements

The regulatory components of AdeABC pump (AdeR and AdeS) were investigated for strains overexpressing *adeB* gene (≥2-fold in real time analysis). PCR and sequencing of *adeS* gene were carried out according to Rumbo et al. (2013). AdeR, AdeN (regulator of AdeIJK pump), and AdeL (regulator of AdeFGH pump) were sequenced with the primers designed in this study using Primer Premier 5.0 software (Supplementary Table S1).

## Results and Discussion

### MLST Profiles of *A. baumannii*

Analysis of Sequence Types (STs) of the 47 *A. baumannii* isolates identified 24 different STs among which seventeen were previously described STs (ST-1, ST-2, ST-7, ST-10, ST-25, ST-32, ST-149, ST-526, ST-575, ST-622, ST-623, ST-625, ST-767, ST-902, ST-905, ST-976, ST-1406). Seven novel STs (ST-1440, ST-1441, ST-1481, ST-1482, ST-1483, ST-1484, ST-1486) were identified and deposited in the MLST database (see text footnote 1). The predominant ST in this study was ST-149 (*n* = 6, 13%). ST profiles of the 47 clinical isolates of *A. baumannii* are summarized in Table 1.

**TABLE 1:** Multilocus Sequence typing (MLST), Clonal Complexes (CC), chromosomal mutations, presence of AAC(6′)-Ib-cr, and Minimum Inhibitory Concentration (MIC) values of ciprofloxacin (CIP), levofloxacin (LVX), and moxifloxacin (MOXI) with or without the presence of efflux pump inhibitors (EPI) (NMP and PAβN) in neonatal septicaemic *Acinetobacter baumannii* (*n* = 47).

Strain number/MLST/clonal complex	Amino acid substitutions within QRDR	Silent mutations	PMQR	MIC in absence and presence of EPIs			Fold change in MIC in presence of EPI		
					
	GyrA/ParC	GyrA and ParC	AAC(6′)-Ib-cr	CIP alone/CIP+NMP/CIP+PAβN	LVX alone/LVX+NMP/LVX+PAβN	MOXI alone/MOXI+NMP/MOXI+PAβN	CIP+NMP/CIP+PAβ N	LVX+NMP/LVX+PAβ N	MOXI+NMP/MOXI+PAβ N
**A_112/ST-32/CC32**	S83L/E84K	Ala193, Pro201 and Leu31, Gly107, Pro109, Lys113, Ala123, Lys124, Ser126, Ala159 Val174, Ala177	+	256/32/256	64/32/32	8/1/4	**8-fold/**NFC	2-fold/2-fold	**8-fold/**2-fold
**A_113/ST-32/CC32**	S83L/E84K	Ala193 and Leu31, Gly107, Pro109, Lys113, Ala123,Ala159,Val174, Ala177	+	64/16/64	4/4/4	4/4/4	**4-fold/**NFC	NFC/NFC	NFC/NFC
A_115/ST-1441/CC126	NM/S80L	Gly114, Ala117,Ala172, Ala193,Pro201 and Ala22, Leu31,Ala48, Gly107,Pro109, Lys113,Ala123, Ala159,Val174	+	32/16/32	2/2/2	2/2/2	2-fold/NFC	NFC/NFC	NFC/NFC
**A_117/ST-10/CC10**	S83L/S80L	Ala193 and Ala22, Leu31,Ala48, Gly107,Pro109, Lys113,Ala123, Ala159,Val174	-	256/64/256	8/2/4	2/1/2	**4-fold/**NFC	**4-fold/**2-fold	2-fold/NFC
A_118/ST-1486/Singleton	S83L/NM	Ala193 and Leu31, Gly107, Pro109, Lys113, Ala123, Iso155, Ala159, Leu166,Thre169, Thre170, Iso172, Ala177	-	8/4/4	0.25/ND/ND	0.125/ND/ND	2-fold/2-fold	ND/ND	ND/ND
A_120/ST-25/CC25	S83L/S80L	Nil and Leu31, Ala48,Ser108, Asp110,Ser114, Ala123,Ala159, Val174	+	32/32/32	4/4/4	2/2/2	NFC/NFC	NFC/NFC	NFC/NFC
A_123/ST-622/CC149	S83L/S80L	Ala172, Ala193 and Leu31, Tyr95, Pro98, Leu99, Iso100, Gly102, Gly107, Pro109 Lys113, Ala123,Ala159, Val174	+	32/32/32	16/16/8	8/4/8	NFC/NFC	NFC/2-fold	2-fold/NFC
**A_124/ST-1440/CC218**	NM/NM	Gly114, Ala117,Ala193 and Leu31, Ala85, Tyr87, Ala123,Ala159, Leu165,Val174	+	256/64/256	0.25/ND/ND	0.125/ND/ND	**64-fold/**NFC	ND/ND	ND/ND
**A_125/ST-2/CC2**	S83L/S80L	Pro160, Ala193,Ala196 and Leu31, Ala48,Gly107, Pro109,Ala123, Ala159, Val174	+	32/16/32	8/8/8	4/0.125/4	2-fold/NFC	NFC/NFC	**32-fold/**NFC
A_126/ST-767/CC216	NM/NM	Gly114, Ala117, Ala193 and Leu31, Ala48,Lys61, Iso100, Ala159, Val174	-	0.25/ND/ND	0.25/ND/ND	0.25/ND/ND	ND/ND	ND/ND	ND/ND
**A_130/ST-575/CC10**	S83L/S80L	Ala193, Ala196 and Leu31, Asp36, Gly107, Pro109, Lys113, Ala123, Iso155, Thre156, Ala159, Leu166, Thre169 Thre170, Iso172, Ala177	-	32/8/16	8/8/8	8/1/2	**4-fold/**2-fold	NFC/NFC	**8-fold/4-fold**
**A_131/ST-902/CC1**	S83L/S80L	Ala193, Pro201 and Ala22, Leu31,Ala48, Gly107,Pro109, Lys113,Ala123, Ala159,Val174	+	64/0.5/32	4/4/4	4/0.25/4	**128-fold/** 2-fold	NFC/NFC	**16-fold/**NFC
**A_132/ST-10/CC10**	S83L, E87Q/S80L	Ala193 and Ala22, Leu31,Ala48, Gly107,Pro109, Lys113,Ala123, Ala159,Val174	-	512/64/128	64/32/32	16/0.25/8	**4-fold/**2-fold	2-fold/2-fold	**64-fold/**NFC
A_133/ST-1406/CC10	S83L/S80L	Ala193 and Leu31, Gly107, Pro109, Lys113, Ala123, Iso155, Thre156, Ala159, Leu166, Thre169, Thre170, Iso172, Ala177	+	32/16/16	8/8/4	8/4/4	2-fold/2-fold	NFC/2-fold	NFC/NFC
A_134/ST-1482/CC149	S83L/S80L	Ala193, Pro201 and Ala22, Leu31,Ala48, Gly107,Pro109, Lys113,Ala123, Ala159,Val174	+	256/256/256	32/16/16	32/16/16	NFC/NFC	2-fold/2-fold	2-fold/NFC
A_135/ST-1481/Singleton	NM/NM	Gly114, Ala117, Iso168, Ala172 And Leu31, Pro98, Leu99, Gly107, Ala123, Gly139, Ala159, Val174	-	1/1/1	1/1/1	0.0625/ND/ND	NFC/NFC	NFC/NFC	ND/ND
**A_136/ST-149/CC149**	S83L/S80L	Ala172, Ala193 and Ala22, Leu31,Ala48, Gly107,Pro109, Lys113,Ala123, Ala159,Val174	-	256/64/64	32/4/16	8/0.125/4	**4-fold/4-fold**	**8-fold/**2-fold	**64-fold/**2-fold
**A_138/ST-905/CC32**	S83L/S80L	Ala193, Pro201 and Ala22, Leu31,Ala48, Gly107, Lys113, Ala123,Ala159,Val174	-	64/16/32	8/2/4	4/4/4	**4-fold/**2-fold	**4-fold/**2-fold	NFC/NFC
A_141/ST-149/CC149	S83L/S80L	Ala172, Ala193 and Ala22, Leu31,Ala48, Gly107, Ala123, Ala159	-	128/64/128	64/32/32	32/16/16	2-fold/NFC	2-fold/2-fold	2-fold/NFC
**A_145/ST-149/CC149**	S83L/S80L	Ala193, Ala196, Pro201 and Ala22, Leu31,Ala48, Gly107,Pro109, Lys113,Ala123, Ala159,Val174	-	256/256/128	64/16/32	32/0.5/16	NFC/2-fold	**4-fold/**2-fold	**64-fold/**NFC
**A_146/ST-1406/CC10**	S83L/S80L	Ala172, Ala193 and Leu31, Tyr95, Arg96, Pro98, Iso100, Gly107, Pro109, Lys113, Ala123, Gly139, Ala159, Val174	+	64/16/32	16/8/8	8/0.125/2	**4-fold/**2-fold	2-fold/2-fold	**64-fold/4-fold**
A_147/ST-1482/CC149	S83L/S80L	Ala172, Ala193 and Ala22, Leu31,Ala48,Gly107, Ala123,Ala159	+	256/256/256	64/32/32	16/16/16	NFC/NFC	2-fold/2-fold	NFC/NFC
**A_149/ST-149/CC149**	S83L/S80L	Ala172, Ala193, Pro201 and Ala22, Leu31,Ala48, Gly107,Pro109, Lys113,Ala123, Ala159,Val174	+	256/32/64	64/16/32	16/4/8	**8-fold/4-fold**	**4-fold/**2-fold	**4-fold/**2-fold
**A_150/ST-10/CC10**	S83L/S80L	Ala193, Pro201 and Leu31,Gly107,Pro109,Lys113,Ala123,Iso155, Thre156,Ala159 Leu166,Thre169,Ala177	+	32/16/32	8/4/4	8/1/4	2-fold/NFC	2-fold/2-fold	**8-fold/**2-fold
A_151/ST-623/CC1	S83L/S80L	Ala193, Pro201 and Ala22, Leu31,Ala48, Gly107, Lys113, Ala123,Ala159,Val174,Tyr209	+	32/16/32	8/4/4	8/8/8	2-fold/NFC	2-fold/2-fold	NFC/NFC
**A_152/ST-623/CC1**	S83L/S80L	Ala193 and Ala22, Leu31,Ala48, Gly107, Lys113, Ala123,Ala159,Val174	+	64/4/64	8/2/8	8/4/4	**16-fold/**NFC	**4-fold/**NFC	2-fold/2-fold
**A_153/ST-623/CC1**	S83L/S80L	Ala193, Pro201 and Ala22, Ala48,Gly107, Pro109,Lys113, Ala123,Ala159, Ala207	+	64/4/64	8/4/4	4/0.125/4	**16-fold/**NFC	2-fold/2-fold	**32-fold/**NFC
**A_155/ST-10/CC10**	S83L/S80L	Ala193, Pro201 and Leu31, Asp36, Gly107, Pro109, Lys113, Ala159, Leu166, Thre169, Thre170, Gly175	-	128/32/128	64/8/32	32/16/16	**4-fold**/NFC	**8-fold/**2-fold	2-fold/2-fold
**A_158/ST-1483/CC10**	S83L/S80L	Ala193 and Ala22, Leu31,Ala48, Gly107, Lys113, Ala123,Ala159,Val174	+	512/64/32	128/32/32	16/16/16	**8-fold/16-fold**	**4-fold/4-fold**	NFC/NFC
**A_159/ST-1483/CC10**	S83L/S80L	Ala193 and Leu31, Gly107,Lys113, Ala123, Iso155, Ala159 Leu166, Thre169,Thre170, Iso172, Ala177	+	512/128/256	128/32/64	32/8/8	**4-fold/**2-fold	**4-fold/**2-fold	**4-fold/4-fold**
**A_160/ST-1/CC1**	S83L/S80L	Ala193, Pro201 and Leu31, Tyr95, Pro98, Iso100, Aspg105, Asp111, Thre121, Gly137, Ala159, Val188, Val189, Ala194	+	128/4/128	32/16/32	16/16/16	**32-fold/**NFC	2-fold/NFC	NFC/NFC
**A_161/ST-149/CC149**	S83L/S80L	Gly170, Ala172 and Ala22, Leu31, Met49, Ser108, Asp110, Pro112, Ser114, Leu125, Val174	+	256/64/128	64/32/32	32/32/32	**4-fold/**2-fold	2-fold/2-fold	NFC/NFC
**A_162/ST-140CC10**	S83L/S80L	Ala193, Pro201 and Leu31, Gly107, Pro109, Phe115, Ala123, Iso155,Aspg167, Ala177	+	64/32/32	32/2/32	8/0.25/2	2-fold/2-fold	**16-fold/**NFC	**32-fold/4-fold**
**A_163/ST-575/CC10**	S83L/S80L	Ala193, Pro201 and Ala22, Leu31,Ala48, Gly107, Pro109, Lys113, Ala123,Ala159, Val174	+	64/16/32	8/8/4	4/0.125/2	**4-fold/**2-fold	NFC/2-fold	**32-fold/**2-fold
**A_166/ST-625/CC513**	S83L/S80L	Ala117, Thre128, Pro201 and Ala22, Leu31,Ala48, Gly107, Pro109, Lys113, Ala123,Ala159, Val174	+	64/32/64	8/4/8	8/0.25/8	2-fold/NFC	2-fold/NFC	**32-fold/**NFC
**A_167/ST-2/CC2**	S83L/S80L	Ala193, Pro201 and Ala22, Leu31,Ala48, Gly107, Pro109, Lys113, Ala123,Ala159, Val174	-	64/64/64	16/8/8	8/0.25/8	NFC/NFC	2-fold/2-fold	**32-fold/**NFC
A_168/ST-149/CC149	S83L/S80L	Ala172, Ala193 and Ala22, Leu31,Ala48, Gly107, Pro109, Lys113, Ala123,Ala159, Val174	-	256/256/256	32/32/32	16/16/16	NFC/NFC	NFC/NFC	NFC/NFC
A_169/ST-976/Singleton	S83L/S80L	Pro201 and Ala22, Leu31,Ala48, Gly107, Pro109, Lys113, Ala123,Ala159, Val174	+	128/128/128	4/4/4	0.5/0.5/0.5	NFC/NFC	NFC/NFC	NFC/NFC
A_170/ST-2/CC2	S83L/S80L	Ala193, Pro201 And Ala22, Leu31,Ala48, Gly107, Pro109, Lys113, Ala123,Ala159, Val174	+	256/128/128	16/16/8	16/16/16	2-fold/2-fold	NFC/2-fold	NFC/NFC
A_171/ST-976/Singleton	S83L/S80L	Pro201 and Leu31, Ala48, Gly107, Phe115, Ala123, Ala159, Val174	+	32/16/64	4/4/4	2/2/2	2-fold/NFC	NFC/NFC	NFC/NFC
**A_172/ST-526/CC2**	S83L/S80L	Ala193 and Leu31, Ala48, Gly107, Phe115, Ala123, Ala159, Val174	+	64/16/64	16/16/16	16/0.125/8	**4-fold/**NFC	NFC/NFC	**128-fold/** 2-fold
**A_173/ST-526/CC2**	S83L/S80L	Ala193, Pro201 and Ala22, Leu31,Ala48, Gly107, Pro109, Lys113, Ala123,Ala159, Val174	-	64/64/64	16/16/16	16/0.125/8	NFC/NFC	NFC/NFC	**128-fold/** 2-fold
A_176/ST-25/CC25	S83L/S80L	Ala193, Pro201 and Leu31, Ala48, Gly107, Phe115, Ala123, Ala159, Val174	+	64/32/64	4/4/4	4/4/4	2-fold/NFC	NFC/NFC	NFC/NFC
**A_177/ST-1484/Singleton**	S83L/S80L	Nil and Leu31, Ala48, Lys61, Iso100, Ala159, Val174	+	64/4/8	4/4/4	4/4/4	**16-fold/8-fold**	NFC/NFC	NFC/NFC
A_178/ST-622/CC149	S83L/S80L	Leu165, Ala193 and Ala22, Leu31,Ala48, Gly107, Pro109, Lys113, Ala123,Ala159, Val174	+	64/32/64	8/8/8	8/8/8	2-fold/NFC	NFC/NFC	NFC/NFC
**A_179/ST-7/CC1**	S83L/S80L	Ala172, Ala193 and Ala22, Leu31,Ala48, Gly107, Pro109, Lys113, Ala123,Ala159, Val174	+	64/32/64	8/2/4	8/4/8	2-fold/NFC	**4-fold/**2-fold	2-fold/NFC
A_180/ST-976/Singleton	S83L/S80L	Pro201 and Leu31, Ala48, Gly107, Phe115, Ala123, Ala159	+	32/32/32	4/4/4	2/2/2	NFC/NFC	NFC/NFC	NFC/NFC

*NM=No mutation detected within QRDR of GyrA or ParC, ND = FQ MIC change in presence of efflux pump inhibitors were not determined for FQ susceptible A. baumannii isolates and written as ND, NFC = No fold change, FQ-resistant A. baumannii isolates which showed ≥4-fold MIC decrease in presence of efflux pump inhibitors were marked as bold and the strain number, sequence types, clonal complexes of these isolates were also indicated as bold.*

**FIGURE 1:**
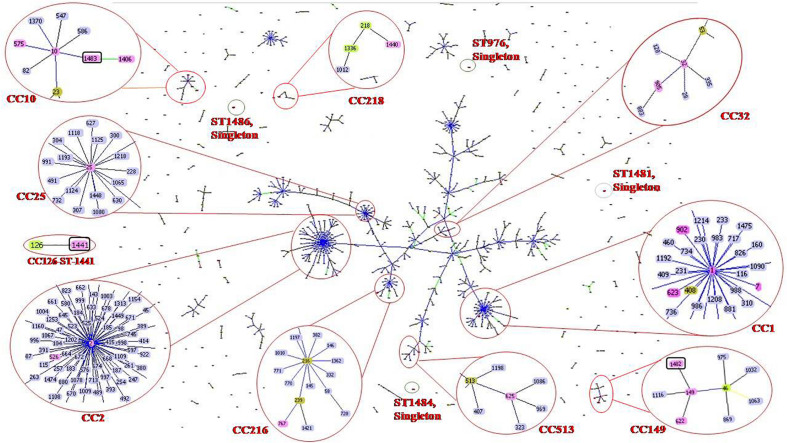
Sequence types (STs) of neonatal septicaemic *Acinetobacter baumannii.* STs of 47 *A. baumannii* are shown in the context of all of the 1507 STs (as per Pasteur analysis) present in the global MLST database (http://www.pasteur.fr/mlst; as of July 2020). The scheme is constructed using the Global optimal eBURST (goeBURST) analysis. The Clonal Complexes (CCs) and STs observed in the present study are enlarged and circled with red color. STs from MLST data base are highlighted with light blue color, the STs identified in the study are highlighted with pink color, founder STs in each CC is highlighted with light green color although few STs (ST-1, ST-2, ST-10, ST-25, ST-32, ST-149) which are identified in this study and also the founder STs, are highlighted with pink. Seven novel STs are identified in the study among which three are related to known CCs, highlighted with pink color and circled with black color. Rest of the novel STs found as singletons is indicated as red dot, circled by black color. Single locus variants (SLVs) of the founder STs in a particular CC are linked by blue or black solid lines (SLV at two different loci for two different STs) and Double locus variants (DLV) are linked by green color solid lines. Numbers of all clonal complexes (CCs) to which the STs were identified in the study are classified.

goeBURST analysis showed that all 47 *A. baumannii* isolates were highly diverse. Isolates were clustered into 10 clonal complexes, **CC1** (ST-1, ST-7, ST-623 and ST-902); **CC2** (ST-2 and ST-526); **CC10** (ST-10, ST-575, ST-1406 and ST-1483); **CC25** (ST-25); **CC32** (ST-32 and ST-905); **CC149** (ST-149, ST-622 and ST-1482); **CC216** (ST767); **CC218** (ST-1440); **CC513** (ST-625); **CC126** (ST-1441). Four STs (ST976, ST1481, ST1484, and ST1486) were found to be singletons since they did not share any homology with the known STs in the data base (Table 1 and Figure 1). Among these singletons, ST-1484 and ST-1486 were novel STs.

### Susceptibility of the Organisms

Ninety-six percent, 92%, and 90% of *A. baumannii* were resistant to ciprofloxacin, levofloxacin, and moxifloxacin, respectively. The majority of isolates (98%) showed high resistance to ciprofloxacin (MIC ≥ 32 mg/L). On the other hand, 80 and 70% of the strains showed MIC of ≥ 8 mg/L for levofloxacin and moxifloxacin, respectively. Only two strains (A_126, A_135) were susceptible to all three FQs tested (Table 1). The strains were highly resistant not only to ciprofloxacin and levofloxacin, but also to 4^th^ generation FQ, moxifloxacin, which is contrary to previous reports (Heinemann et al., 2000; Spence and Towner, 2003; Liu et al., 2012). This indicates that even the higher generation FQ, such as moxifloxacin is not active against *A. baumannii*. This is probably a rare finding in case of neonatal septicaemic *A. baumannii*. The high FQ resistance among *A. baumannii* may be due to excessive use or exploitation of these agents worldwide for different therapeutic purposes or the presence of multiple mechanisms that have been found to be functional in *A. baumannii*. Since most of the *A. baumannii* were resistant to ciprofloxacin (96%), levofloxacin (92%), and moxifloxacin (90%), thus susceptibility of the organisms toward FQs was not associated with a particular ST-type or clonal complex.

**FIGURE 2:**
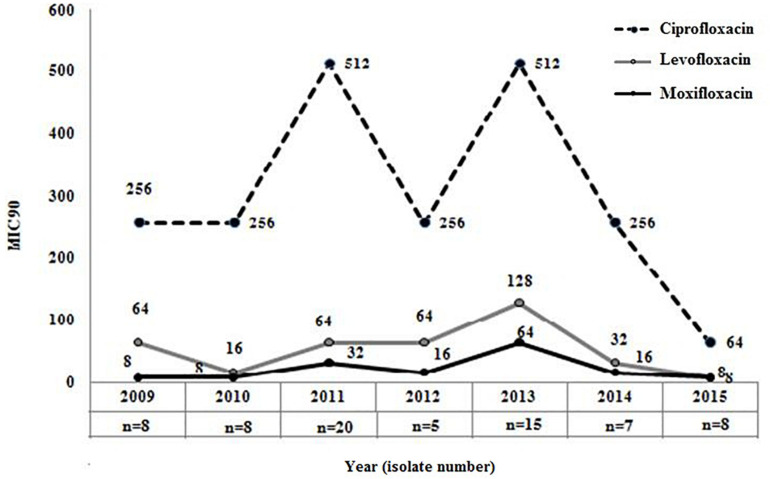
Distribution of MIC_90_ for three clinically important fluoroquinolones among *Acinetobacter baumannii*. MIC_90_ of ciprofloxacin (2^nd^ generation), levofloxacin (2^nd^ generation), and moxifloxacin (4^th^ generation) was determined by broth microdilution method among all septicaemic *A. baumannii* (*n* = 47) isolated during 2009–2015.

The overall (2009−2015) MIC_90_ values for the different FQs were as follows: ciprofloxacin, 256 mg/L; levofloxacin, 64 mg/L; and moxifloxacin, 32 mg/L, respectively. Comparison of MIC_90_ values for FQs also indicated that though levofloxacin or moxifloxacin MIC in each year was ≥ 4-fold lower in comparison to the MIC of ciprofloxacin but 90% of the strains in each year were resistant to these three FQs (Figure 2). This again indicated that most of the septicemic *A. baumannii* were highly resistant to older and newer clinically important FQs.

Besides fluoroquinolone resistance, organisms showed resistance to other antimicrobials such as ceftazidime (81%), cefepime (81%), meropenem (77%), imipenem (79%), aztreonam (94%), amikacin (66%) and colistin (9%). No resistance was detected for minocycline.

### Mutations Within QRDR of GyrA and ParC

The major mutations that were identified in this study were S83L (93%) and S80L (96%) within the QRDRs of GyrA and ParC in FQ-resistant *A. baumannii* (FQRAB) (Table 1). Though the information is not new, however, it collaborates with other earlier studies and underscores the importance of chromosomal mutations in FQ resistance. Mutations in the QRDRs of both GyrA/ParC alter the three-dimensional structure of the target protein. Alteration of the target protein structure reduces the affinity of quinolones for the enzyme-DNA complex (Hooper and Jacoby, 2015; Kivata et al., 2019). In this study, no amino acid sequence changes were observed at the other “hot spots” (Gly-81, and Ala-84) within the GyrA as reported previously (Valentine et al., 2008). Consistent with the literature (Vila, 2002), the FQRAB (98%, 44/45) which possessed both *gyrA* and *parC* mutations, had a ciprofloxacin MIC ≥ 32 mg/L. Isolate A_132 with ciprofloxacin MIC 512 mg/L, possessed more than one mutation (S83L and E87Q) within GyrA and also possessed S80L within ParC. On the other hand, isolate A_118 showed only S83L conversion within GyrA but no mutation within ParC and had a MIC of CIP 8 mg/L. This isolate was susceptible to levofloxacin and moxifloxacin. Similarly, another isolate A_115 with ciprofloxacin MIC 32 mg/L, showed only S80L conversion within ParC but no mutation within GyrA. This isolate was resistant to levofloxacin and moxifloxacin (MIC 2 mg/L) (Table 1). This indicated that a single *gyrA* mutation is sufficient to cause clinically significant levels of ciprofloxacin resistance (Maleki et al., 2014; Ardebili et al., 2015) but mutation within *parC* gene or more than one mutation within *gyrA* is necessary for high-level ciprofloxacin resistance in *A. baumannii* as reported previously (Vila, 2002; Spence and Towner, 2003). Moreover, S80L mutation within ParC is also necessary for levofloxacin and moxifloxacin resistance (Spence and Towner, 2003).

Two other FQRAB (A_112 and A_113) had E84K mutation within ParC instead of S80L. These mutations (E87Q and E84K) had previously been reported to be associated with FQ resistance in *A. baumannii* (Valentine et al., 2008; Park et al., 2011). Alteration of Glu-84 to Lys within ParC implies that replacement of acidic amino acid with basic amino acid at position 84 may change the structural arrangement of the active site of ParC and its contact with surrounding amino acids which in turn make FQs incapable to bind to the enzyme. Several other mutations within GyrA (Gly81Val, Ala84Pro, Gly81Cys, Gly81Asp, Ser97Thr) and ParC (Lys59Gln, Gly78Cys, Ser80Trp, ser80Tyr, Ala85Pro), associated with FQ resistance in FQRAB, were reported by other studies but such mutations were not detected in any of the study isolates (Hamouda and Amyes, 2004; Chiu et al., 2010; Park et al., 2011; Hongbo et al., 2013; Ardebili et al., 2015). Isolate A_124 showed high MIC for ciprofloxacin (256 mg/L) but had no mutations within ParC or GyrA. This isolate showed overexpression of AdeB efflux pump. Since both mutations within GyrA (S83L) and ParC (S80L) were observed in diverse STs, thus chromosomal mutations responsible for FQ resistance were not associated with any particular ST or clonal complex, rather detected among 10 clonal complexes found in this study. Silent mutations within *gyrA* and *parC* were also observed (Table 1). In the case of *parC*, all isolates possessed more than seven different silent mutations. The numbers of silent mutations within *parC* gene was higher than *gyrA* gene (47 vs. 12) as also observed in previous studies (Sun et al., 2015). The function of these mutations is still unknown.

### Prevalence of PMQRs

Among the PMQRs investigated in this study, only *aac(6′)-Ib-cr* was detected in 74% of ciprofloxacin-resistant *A. baumannii* (Table 1). None of the *A. baumannii* showed the presence of *qnr* genes. It has previously been reported that strains possessing this enzyme remain susceptible as acetylation of FQ with AAC(6′)-Ib-cr reduces drug activity and confers a low level of FQ resistance (Rodríguez-Martínez et al., 2016). But this enzyme may create an environment which facilitates topoisomerase mutations (Frasson et al., 2011). In this study, AAC(6′)-Ib-cr-possessing FQRAB isolates also possessed chromosomal mutations and/or showed overexpression of efflux pumps (discussed below). Thus, isolates are highly resistant to FQs.

### Effects of EPIs on FQ Resistance

None of the isolates showed the bactericidal effect of EPIs at a concentration of 50 mg/L. This confirms that the reduction of MIC for FQs in presence of EPIs was due to the presence of efflux pumps. Reduction in MIC with NMP was observed for ciprofloxacin (4−128-folds), levofloxacin (4−16-fold), and moxifloxacin (4−128-fold) among 48, 25, and 44% of FQRAB, respectively. On the other hand, PAβN also decreased the MIC of FQs to some extent [ciprofloxacin (4−16-folds) in 11% of FQRAB, levofloxacin (4-fold) in 2% of FQRAB, moxifloxacin (4-fold) in 12% of FQRAB]. Of the two EPIs used, NMP was more active in reduction of FQ MIC than PAβN. This result is in agreement with a previous report suggesting that activities of these two compounds were dissimilar (Pannek et al., 2006). This is consistent with the view that different antibiotics may have different binding sites on the pump with which the EPI (here NMP or PAβN) might interact in a variable manner (Elkins and Nikaido, 2002).

Overall, 63% (*n* = 29) of FQRAB showed reduction of MICs (4−128-fold) for FQs in presence of EPIs and 37% of the FQRAB were not affected by EPIs, establishing the fact that FQ resistance is not solely due to the participation of efflux pumps in some of the strains (Table 1). The strains which showed participation of efflux pumps in FQ resistance belonged predominantly to CC1 (5 out of 6 isolates), CC2 (4 out of 5 isolates), CC10 (10 out of 11 isolates), and CC32 (all 3 isolates of this group). Very few isolates belonging to CC149 (4 out of 10 isolates), CC218 (*n* = 1), and CC513 (*n* = 1) also showed participation of efflux pumps in presence of EPI (Table 2).

**TABLE 2:** Expression levels (fold change) of RND-family (AdeB, AdeJ, AdeG) and MATE-family efflux pumps (AbeM) detected by reverse transcriptase quantitative PCR (RT-qPCR) in *Acinetobacter baumannii* that showed reduction of MIC for fluoroquinolones in presence of efflux pump inhibitors.

Strain No	MLST/Clonal complex	*adeB* fold change	*adeJ* fold change	*adeG* fold change	*abeM* fold change
A_112	ST-32/CC32	**3.6**	**2.1**	1.2	0.2
A_113	ST032/CC32	**3.7**	**4.1**	1.1	**2**
A_117	ST-10/CC10	ND	**2.1**	**5.6**	ND
A_124	ST-1440/CC218	**3.03**	ND	ND	0.29
A_125	ST-2/CC2	**7.0**	**2.8**	**7.7**	**2.69**
A_130	ST-575/CC10	0.06	**2.9**	0.14	0.6
A_131	ST-902/CC1	**34**	**2**	0.73	1.4
A_132	ST-10/CC10	0.044	**3.5**	1.3	**4.47**
A_136	ST-149/CC149	**5**	ND	ND	0.7
A_138	ST-905/CC32	**8.65**	**3.1**	0.8	**2.0**
A_145	ST-149/CC149	**12.7**	0.7	0.6	0.8
A_146	ST-1406/CC10	**3**	0.1	ND	0.1
A_149	ST-149/CC149	**2.2**	0.2	0.7	0.4
A_150	ST-10/CC10	ND	**3.79**	ND	0.49
A_152	ST-623CC1	1.5	1.2	0.4	**5.9**
A_153	ST-623/CC1	1.1	0.5	**13.9**	**7.5**
A_155	ST-10/CC10	**5.4**	**3.1**	0.3	0.5
A_158	ST-1483/CC10	**22**	1.5	**2.5**	1.1
A_159	ST-1483/CC10	ND	**2.4**	ND	1.2
A_160	ST-1/CC1	0.1	ND	ND	0.12
A_161	ST-149/CC149	**21.5**	0.1	0.8	0.9
A_162	ST-1406/CC10	**17.5**	**2.8**	0.03	0.2
A_163	ST-575/CC10	**20**	**2.8**	0.007	0.6
A_166	ST-625/CC513	**11.0**	**10.7**	**3.2**	1.05
A_167	ST-2/CC2	**25.3**	**2.5**	**2.8**	1.7
A_172	ST-526/CC2	**9.8**	0.5	1.8	**4**
A_173	ST-526/CC2	**18.35**	**10.64**	**17.4**	1.097
A_177	ST-1484 (singleton)	0.021	0.757	0.383	**25**
A-179	ST-7/CC1	**15.0**	0.6	0.25	1.1

*RND, Resistance Nodulation Division family; MATE, Multidrug and Toxin Extrusion family; MIC, Minimum Inhibitory Concentration; ND, No Detection. The relative gene expression (△CT) for pump genes transcripts was calculated against that for the 16S rRNA gene, [△CT of test gene= CT of test gene − CT of 16S] and the △△CT was calculated against that for the ciprofloxacin-susceptible strain, A. baumannii ATCC 19606 (expression = 1), which served as the control. Relative expression levels of pump genes were calculated by the 2^−ΔΔCT^ method. Expression level of pump gene which was ≥2-fold higher in comparison to the control strain A. baumannii ATCC 19606 (expression = 1), indicated as bold and considered as overexpression of the pump.*

### Overexpression of Efflux Pumps

In this study, four different efflux pump genes were investigated for each isolate and such simultaneous assessment of multiple pumps had not been carried out before. Earlier studies reported overexpression of either one pump (AdeABC) or two pumps together (AdeABC, AeIJK) (Chiu et al., 2010; Park et al., 2011; Lopes and Amyes, 2013; Ardebili et al., 2014). Overexpression of *adeB* gene was detected among 70% (21/30) of *A. baumannii* strains which showed ≥4-fold decrease in MICs for FQs in presence of EPIs. This was followed by *adeJ* (53%, *n* = 16), *abeM* (30%, *n* = 9), and *adeG* (23%, *n* = 7) (Table 2). Significantly higher numbers of *A. baumannii* showed overexpression of *adeB* in comparison to *adeG* (Fisher’s exact test; *p* = 0.0041) and *adeM* (Fisher’s exact test; *p* = 0.0006). However, no statistically significant differences were found between the numbers of strains overexpressing *adeB* and *adeJ* (Fisher’s exact test; *p* = 0.1872). However, the relative expression level of *adeB* was highest (2.2−34-fold) among all pumps tested. The other target efflux pump genes *adeJ*, *adeG*, and *abeM* showed expression levels between 2.1−10.7-fold, 2−17.4-fold, and 2−25-fold, respectively (Table 2). This indicated that though the involvement of *adeB* was highest in FQ resistance, overexpression of other pumps cannot be neglected. Since AdeIJK pump is intrinsic to *A. baumannii*, thus it was detected among large numbers of strains but in comparison to *adeB*, the relative fold change of *adeJ* was low supporting the fact that overexpression of AdeIJK is toxic to *A. baumannii* (Damier-Piolle et al., 2008). In three out of 30 isolates (A_117, A_150, and A_159), *adeB* could not be amplified. On the other hand, in five out of 30 isolates (A_130, A_132, A_152, A_153, and A_177), overexpression of AdeABC was not detected. In these five isolates, either AdeRS could not be detected or mutations were absent within AdeRS. It has previously been reported that the loss of *adeRS* or *adeB* could significantly modify the transcriptional level of AdeABC efflux pump of *A. baumannii* resulting in a decreased expression of *adeABC* (Richmond et al., 2016). In these isolates, an association of overexpression of other pumps (*adeJ* or *adeG* or *abeM*) with elevated FQ MICs is thus likely (Table 2). One isolate (A_160) did not show overexpression of any of the efflux pumps studied here in spite of FQ MIC decrement in presence of EPI indicating involvement of other efflux pumps as five uncharacterized RND pumps have been reported in *A. baumannii* (Nowak et al., 2015). In this study, ≥ 2 different pumps were overexpressed simultaneously among 38% of FQRAB (17/45).

### Correlation Between Fluoroquinolone MIC and Different Resistance Mechanisms

An attempt was made to correlate the FQ MIC values with the three different resistance mechanisms studied here: chromosomal mutations, over expression of efflux pumps, and presence of *aac(6′)-Ib-cr*.

Presence of these three mechanisms simultaneously was detected among 33% of FQRAB and the FQ MIC for three drugs was as follows: ciprofloxacin MIC (64−512) mg/L, levofloxacin MIC (4−128) mg/L, and moxifloxacin MIC (4−64) mg/L. FQRAB (30%) that did not express efflux pumps but where the other two mechanisms were detected exhibited ciprofloxacin MIC (32−256) mg/L, levofloxacin MIC (2−64) mg/L, and moxifloxacin MIC (0.5−32) mg/L. Decreased FQ MIC (≥2-fold) in these strains clearly indicated the role of efflux pumps in FQ resistance. However, 13% of FQRAB did not possess AAC(6′)-Ib-cr, but showed chromosomal mutations along with overexpression of pumps and had ciprofloxacin MIC (64−512 mg/L). AAC(6′)-Ib-cr can reduce the activity of ciprofloxacin by acetylation but was not able to acetylate moxifloxacin and levofloxacin (Robicsek et al., 2006). The ciprofloxacin MIC in the study isolates with or without AAC(6′)-Ib-cr could not be assessed as the other two mechanisms were also present. Mutations only in GyrA and ParC were detected in 7% of strains without the involvement of efflux pumps and *aac(6′)-Ib-cr*. These isolates showed ≥8-fold lower FQ MIC as follows: ciprofloxacin MIC (8−256) mg/L, levofloxacin MIC (0.25−64) mg/L, moxifloxacin MIC (0.125−32) mg/L (Supplementary Table S2).

When three mechanisms work together in isolates, it is difficult to delineate the contribution of each mechanism. However, the study showed that both chromosomal mutations and efflux pumps are important as the absence of pumps is reflected in the lower MIC values. Strains with all three mechanisms showed high FQ MIC.

### Analysis of Regulators of RND Family Efflux Pumps

To further assess the mechanism of *adeB, adeJ*, and *adeG* overexpression, the protein sequences of the respective pump regulators AdeRS, AdeN, and adeL were studied. These sequences were compared with the four *A. baumannii* reference strains (*A. baumannii* AYE, ATCC 17978, ATCC 19606, and *A. baumannii* ACICU) in order to exclude polymorphisms.

The AdeABC efflux pump is controlled by the AdeRS two-component regulatory system (Yoon et al., 2013). AdeS is the sensor histidine kinase (HK) that autophosphorylates at an internal histidine. The phosphate group is then transferred to an aspartate residue of the cytoplasmic response regulator which acts as a transcriptional activator (AdeR). Phosphorylated AdeR binds to an intercistronic space (ICS), located between the promoter and coding sequences of adeABC. This binding prevents the transcription of adeABC mRNA. Thus, binding of AdeR to ICS control the expression of AdeABC operon (Chang et al., 2016).

In AdeR, four different amino acid substitutions (F132S, I175L, L227I, and I228V) were detected (Table 3). The substitutions were present either as single substitution or in combinations. None of these substitutions were detected in the reference strains. The substitution F132S (*n* = 1) was located in the signal receiver domain of AdeR. Mutation in this domain may change the interactions between the AdeS and AdeR and prevent the subsequent binding to intercistronic space (ICS) leading to AdeABC overexpression (Yoon et al., 2013). Furthermore, substitutions I175L (*n* = 2), L227I (*n* = 2), and I228V (*n* = 1), located in the effector domain again prevent the binding of AdeR to ICS and cause overexpression of AdeABC which subsequently leads to FQ resistance (Table 3). Two substitutions of AdeR (I120 and A136V) were detected in most of the AdeABC-overexpressing strains (11/21). These two substitutions were considered as silent polymorphisms as they were also detected in the reference strain *A. baumannii* ACICU (Supplementary Table S3; Gerson et al., 2018).

**TABLE 3:** Amino acids substitutions in AdeRS two component system in fluoroquinolone-resistant *Acinetobacter baumannii* isolates overexpressing AdeABC efflux pump.

		Amino acid at the indicated position*									
		AdeS (360 aa)						AdeR (247 aa)			
Different mutational patterns in AdeRS (No. of isolates in each pattern)	Isolate numbers (MLST/CC)	HAMP (82−138)	DHp (138−246)		CA (246−360)			REC (1−140)	Output domain (141−247)		
		121	186	188	255	257	322	132	175	227	228
		E	G	S	V	I	L	F	I	L	I
Type 1 (*n* = 1)	A_112 (ST-32/CC32)				I				L	I	
Type 2 (*n* = 1)	A_113 (ST-32/CC32)			F						I	
Type 3 (*n* = 1)	A_124 (ST-1440/CC218)		V								V
Type 4 (*n* = 1)	A_138 (ST-905/CC32)		V		I				L		
Type 5 (*n* = 1)	A_166 (ST-625/CC513)					V		S			
Type 6 (*n* = 1)	A_146 (ST-1406/CC10)	K						No amino acid substitutions			
Type 7 (*n* = 2)	A_136 (ST-149/CC149), A_149 (ST_149/CC149)			F	I			No amino acid substitutions			
Type 8 (*n* = 7)	A_125 (ST-2/CC2), A_162 (ST-1406/CC10), A_163 (ST-575/CC10), A_167 (ST-2/CC2), A_172 (ST-526/CC2), A_173 (ST-526/CC2), A_179 (ST-7/CC1)		V					No amino acid substitutions			
Type 9 (*n* = 1)	A_161 (ST-149/CC149)				I			No amino acid substitutions			
Type 10 (*n* = 2)	A_131 (ST-902/CC1), A_158 (ST-1483/CC10)			F	I			No amino acid substitutions			
Type 11 (*n* = 1)	A_145 (ST-149/CC149)			F	I		F	No amino acid substitutions			
Type 12 (*n* = 1)	A_155 (ST-10/CC10)	K					F	No amino acid substitutions			

aa, amino acids; CA, catalytic and ATP-binding domain; DHp, dimerization and histidine-containing phosphotransfer domain; HAMP, histidine kinase, adenylylcyclase, methyl-accepting protein, and phosphatase linker domain; REC, receiver domain; CC, Clonal Complex. * The AdeR and AdeS amino acid sequences of A. baumannii ACICU, A. baumannii AYE, ATCC 17978, and ATCC 19606 were used as reference strains for comparison. Amino acid substitutions are indicated using the one-letter code.

Six different amino acid substitutions (E121K, G186V, S188F, V255I, I257V, L322F) were detected in AdeS (Table 3). None of these substitutions were detected in the reference strains. Of these substitutions, G186V (*n* = 9) was located in the α-helix of the DHp domain and has previously been reported (Table 3). Mutation in this domain could prevent the phospho-transfer to AdeR. Thus, inability of AdeR to bind to the ICS region leads to lack of control over the expression of AdeABC efflux pump, leading to FQ resistance (Yoon et al., 2013; Sun et al., 2016). Substitutions E121K (*n* = 2) and S188F (*n* = 6) were located in the HAMP domain and C-terminal end of the DHp domain of histidine kinase, respectively. Amino acid substitutions in the HAMP domain of AdeS protein disrupt transmembrane signal transduction and have been suggested to be associated with constitutive phenotypes and overexpression of AdeABC effux pump (Yoon et al., 2013). In addition, substitutions V255I (*n* = 6), I257V (*n* = 1), and L322F (*n* = 2) were located in the catalytic domain. Mutations in this domain could affect ATP binding leading to prevention of autophosphorylation of AdeS which simultaneously prevent the phosphotransfer to AdeR. Thus, AdeR does not bind to the ICS region, leading to hyperexpression of AdeABC efflux pump (Table 3; Yoon et al., 2013). Several common polymorphisms within AdeS (V27I, V32I, A94V, L172P, F214L, D227H, N268H, S280A, Q281D, Q299R, Y303F, I331V, and Q339K) were also detected in the reference strains and in some study isolates not overexpressing AdeABC (Supplementary Table S3). These changes were considered to be silent polymorphisms and not associated with overexpression of the pump and were excluded (Yoon et al., 2013). In this study, mutations within AdeR and AdeS were not found to be associated with any particular clonal complex but were rather found within diverse STs belonging to different clonal complexes (CC1, CC2, CC10, CC32, CC149, CC218, CC513) (Table 3).

In AdeN (regulator of AdeIJK), amino acid substitutions K15E (*n* = 1), P16T (*n* = 2), Q17P (*n* = 1), L26S (*n* = 1), F64I (*n* = 4), and M174T (*n* = 3) were detected in different combinations among 12 isolates while in AdeL (regulator of AdeFGH), substitutions L34F (*n* = 1), I50F (*n* = 2), L38V (*n* = 1), T46D (*n* = 1), and D107V (*n* = 2) were detected in different combinations within three isolates. These amino acid substitutions seemed to be associated with FQ resistance as these mutations were not present in the reference strains (Table 4). Premature stop codons had been detected in two isolates at codon position 13 and 175 within the protein sequence of AdeN. Amino acid substitutions within AdeN were predominantly detected among *A. baumannii* belonging to clonal complex CC10 and CC32 whereas amino acid substitutions within AdeL detected in only three isolates belonged to CC1, CC2, and CC10 (Table 4). Few of the isolates (*n* = 4, Table 4) showed overexpression of AdeIJK and AdeFGH but did not possess any mutations within AdeN and AdeL regulators, respectively. Furthermore, disruption of *adeRS* or *adeN* by insertion of IS elements and nucleotide deletions or insertion within the regulators were not evident in any of the study isolates as had been reported previously (Park et al., 2011; Gerson et al., 2018). The amino acid substitutions detected in the three regulators, has not been described previously, future studies should be carried out to know the role of these mutations and their association with FQ resistance phenotype.

**TABLE 4:** Amino acid substitutions in AdeN and AdeL in fluoroquinolone-resistant *Acinetobacter baumannii* (FQRAB) overexpressing AdeIJK and AdeFGH efflux pumps, respectively.

RND-family efflux pumps	Regulators of the respective pumps	No of FQRAB overexpressing the efflux pumps	Mutations within the regulators (No of FQRAB with overexpression of the pump and possessed the changes)	Isolate numbers (MLST/CC)
AdeJ	AdeN	16	K15E (*n* = 1)	A_167 (ST-2/CC2)
			P16T, Q17P (*n* = 1)	A_112 (ST-32/CC32)
			P16T (*n* = 1)	A_113 (ST-32/CC32)
			L26S, F64I (*n* = 1)	A_159 (ST-1483/CC10)
			F64I (*n* = 3)	A_117 (ST-10/CC10), A_150 (ST-10/CC10) A_155 (ST-10/CC10)
			M174T (*n* = 3)	A_130 (ST-575/CC10), A_162 (ST-1406/CC10), A-163 (ST-575/CC10)
			Stop codon at codon numbers 13 and 175 (*n* = 2)	A_132 (ST-10/CC10), A_138 (ST-905/CC32)
			No amino acids substitution (*n* = 4)	A_125 (ST-2/CC2), A_131 (ST-902/CC1), A_166 (ST-625/CC513), A_173 (ST-526/CC2)
AdeG	AdeL	7	I50F (*n* = 1)	A_125 (ST-2/CC2)
			L34F, I50F, D107V (*n* = 1)	A_153 (ST-623/CC1)
			L38V, T46D, D107V (*n* = 1)	A_158 (ST-1483/CC10)
			No mutations detected (*n* = 4)	A_117 (ST-10/CC10), A_166 (ST-625/CC513), A_167 (ST-2/CC2), A_173 (ST-526/CC2)

## Conclusion

FQ-resistance has been studied earlier by several authors highlighting different mechanisms simultaneously in gram-negative organisms (Oethinger et al., 2000; Ge et al., 2005; Lopes and Amyes, 2013). However, none of these studies analyzed the cumulative effect of multiple mechanisms of FQ resistance among diverse STs of neonatal septicemic *A. baumannii*. Moreover, as the testing of newer FQs continues, there is no comprehensive data regarding the resistance to higher generation FQs such as moxifloxacin available from India in spite of high antimicrobial resistance. This is most concerning as 90% of *A. baumannii* isolated from neonates in this study were resistant to moxifloxacin.

This study showed the diversity of the strains in this NICU during the study period as 24 different STs were detected among 47 *A. baumannii* with seven novel sequence types: ST-1440, ST-1441, ST-1481, ST-1482, ST-1483, ST-1484, and ST-1486. Our approach also revealed chromosomal mutations [both GyrA (S83L) and ParC (S80L)] were the predominant mechanism associated with FQ resistance as it was detected in > 90% of FQRAB analyzed. Previous studies have reported several other mutations associated with FQ resistance within GyrA and ParC of FQRAB which were not evident in this study. The second important mechanism associated with FQ resistance was overexpression of efflux pumps. Earlier studies reported overexpression of either one pump (AdeABC) or simultaneous overexpression of two pumps together (AdeABC, AeIJK). In this study, four different efflux pumps were investigated for each isolate and such simultaneous assessment of multiple pumps had not been carried out before. Simultaneous overexpression of ≥2 different pumps was detected among 38% of *A. baumannii*. Though the m-RNA transcription level of *adeB* was highest but other pumps were also found to be associated with FQ resistance. In this study, lower MIC of FQ (≥8-folds) was detected among the FQRAB which showed only chromosomal mutations in comparison to the strains where both chromosomal mutations and overexpression of efflux pumps were detected simultaneously. This indicated the importance of efflux pumps in FQ resistance. Among the PMQRs investigated in this study, only AAC(6′)-Ib-cr was detected in a substantial proportion of FQRAB. Though, presence of this enzyme confers lower level of resistance to ciprofloxacin but they facilitate the generation of GyrA/ParC mutations. In this study, AAC(6′)-Ib-cr-possessing FQRAB also had chromosomal mutations and/or showed overexpression of efflux pumps, exhibiting high resistance to FQs. Overall, presence of chromosomal mutations was evident among all 10 clonal groups (CC1, CC2, CC10, CC25, CC32, CC126, CC149, CC216, CC218, and CC513) detected in this study. On the other hand, participation of efflux pumps was predominantly detected among the isolates belonging to CC1, CC2, CC10, and CC32. In contrast to earlier studies which reported disruption of *adeRS* or *adeN* by insertion of IS elements, nucleotide deletions or insertion within the pump regulators, this study revealed amino acid substitutions in the regulatory proteins of RND-pumps. It shows that *A. baumannii* isolated from different geographical locations exhibit differences in mutational patterns in the regulators. Mutations within AdeRS detected among different STs belonged to different clonal complexes and were not associated to a particular clonal complex. On the other hand, mutations within AdeN predominantly belonged to CC10 and CC32.

Interpretation of susceptibility patterns by studying the cumulative effect of various mechanisms is not an easy task. This study noted high variability of FQ susceptibility among FQRAB in spite of possessing the same set of resistance mutations in GyrA, ParC, and, efflux pump regulators. This variability might be due to other factors such as the reduction in outer membrane porin diffusion channels, or mutations in genes encoding regulatory proteins of the porin genes or mutations in efflux pump structural genes or involvement of other efflux pumps or even mutations in *gyrB*/*parE* that were not analyzed in this study (Hamouda and Amyes, 2006; Hooper and Jacoby, 2015). Further, the study showed that antibiotic resistance is dynamic, and changes geographically. An understanding of susceptibility patterns of MDR *A. baumannii* toward higher generation fluoroquinolones such as moxifloxacin in developing countries is important to optimize therapeutic strategies accordingly.

## Data Availability Statement

The raw data supporting the conclusions of this article will be made available by the authors, without undue reservation.

## Ethics Statement

The study protocol was carefully reviewed and approved by the Institutional Ethics Committee of the ICMR-National Institute of Cholera and Enteric Diseases (Indian Council of Medical Research) (No. A-1/2016-IEC, dated 3^rd^ May 2016). Individual informed consent was not required as the study was carried out on *Acinetobacter baumannii* strains archived at ICMR-NICED. These strains were isolated from neonates during the course of routine diagnosis of sepsis and did not pose any additional risks to the patients.

## Author Contributions

SR, SC, and AB performed the experiments. SR participated in experimental design, manuscript writing, and interpretation of data. PC and BS helped in acquisition of clinical strains. SD helped in drafting of manuscript. SB conceived the study, participated in data analysis, oversaw the project, and was involved in manuscript writing. All authors contributed to the article and approved the submitted version.

## Conflict of Interest

The authors declare that the research was conducted in the absence of any commercial or financial relationships that could be construed as a potential conflict of interest.

## Footnotes

1 https://pubmlst.org/abaumannii/
