# Supplementary material for: Overexpression of Efflux Pumps, Mutations in the Pumps’ Regulators, Chromosomal Mutations, and AAC(6′)-Ib-cr Are Associated With Fluoroquinolone Resistance in Diverse Sequence Types of Neonatal Septicaemic Acinetobacter baumannii: A 7-Year Single Center Study
Source: Front Microbiol. 2021 Mar 11;12:602724. doi: 10.3389/fmicb.2021.602724 (PMC7990795; doi:10.3389/fmicb.2021.602724)
Supplement: Supplementary file 1 [file Table_1.doc]

| **Gene** | **Primer** | **Sequence (5’-3’)** | **Size (bp)** | **T_m_** | **Position** | **Reference** |
| --- | --- | --- | --- | --- | --- | --- |
| *abeB* | abeB-F  abeB-R | CTTGCATTTACGTGTGGTGT  GCTTTTCTACTGCACCCAAA | 168 | 58 | 2907–2926  3075–3056 | Coyne *et al.* |
| *abeJ* | abeJ-F  abeJ-R | GGTCATTAATATCTTTGGC  GGTACGAATACCGCTGTCA | 221 | 58 | 1142–1160  1363–1345 | Coyne *et al.* |
| *abeG* | abeG-F  abeG-R | GTGTAGTGCCACTGGTTACT  ATGTGGGCTAGCTAACGGC | 203 | 58 | 2969-2989  3171-3152 | This study |
| *abeM* | abeM-F  abeM-R | CGGTTTCTAGGTGGAGTGGT  ACCGAAACGACGTGACATTC | 115 | 55 | 6-24  120-100 | This study |
| *16s rRNA* | 16s-F  16s-R | TCCAGGTGTAGCGGTGAAAT  CCCCAACGGCTAGTAGACAT | 161 | 55or 58 | 666-686  826-806 | This study |
| *adeR* | adeR-F  adeR-R | AGATGACTACGATATTGGCGAC  AGTCTATATCCCACGCCACG | 654 | 58 | 56-78  710-690 | This study |
| *adeS* | adeS-F  adeS-R | TATGAAAAGTAAGTTAGGAAT  TTAGTTATTCATAGAAATTTT | 1072 | 50 | 1-19  1073-1053 | Coyne *et al.* |
| *adeN* | adeN-F  adeN-R | TGCATGATCCAGTCCTTGAGT  CTTCTGTCTCATTGGGTGGG | 594 | 55 | 2-23  595-575 | This study |
| *adeL* | adeL-F  adeL-R | GACTACAACCATTCAGGC  GAGCGTATAAGCTTCAGC | 925 | 50 | 80-98  1005-987 | This study |
| *gyrA* | gyrA-F  gyrA-R | GGAAATCCGACCGATTGCC  CGAGATATTCGGATTGTCAGC | 620 | 54 | 12-30  606-582 | Hujer *et al.* |
| *parC* | parC-F  parC-R | ATGGAAGATAAGCTGACTATG  GTTGGTAAATCCGGAGC | 680 | 54 | -18-3  662-645 | Hujer *et al.* |

**TABLE S1. Oligonucleotides used in Reverse transcriptase quantitative PCR (RT-qPCR) and sequencing**
