# Supplementary material for: Overexpression of Efflux Pumps, Mutations in the Pumps’ Regulators, Chromosomal Mutations, and AAC(6′)-Ib-cr Are Associated With Fluoroquinolone Resistance in Diverse Sequence Types of Neonatal Septicaemic Acinetobacter baumannii: A 7-Year Single Center Study
Source: Front Microbiol. 2021 Mar 11;12:602724. doi: 10.3389/fmicb.2021.602724 (PMC7990795; doi:10.3389/fmicb.2021.602724)
Supplement: Supplementary file 2 [file Table_2.doc]

| **Chromosomal mutations** | **Over expression of pump** | **Presence of *aac6’Ib-cr*** | **CIP MIC range (mg/L)** | **LVX MIC range (mg/L)** | **MOXI MIC range (mg/L)** | **Number of isolates (%)** |
| --- | --- | --- | --- | --- | --- | --- |
| + | + | + | CIP: (64-512) | LVX: (4-128) | MOXI: (4-64) | 15 (33%) |
| + | - | + | CIP: (32-256) | LVX: (2-64) | MOXI: (0.5-32) | 14 (30%) |
| + | + | - | CIP: (64-512) | LVX: (8-64) | MOXI: (2-32) | 6 (13%) |
| + | - | - | CIP: (8-256) | LVX: (0.25-64) | MOXI: (0.125-32) | 3 (7%) |
| - | + | + | CIP: (256) | LVX: (0.25) | MOXI: (0.125) | 1 (2%) |

Table S2. Correlation of ciprofloxacin (CIP, levofloxacin (LVX) and moxifloxacin (MOXI) MICs with chromosomal mutations and / or overexpression of efflux pumps and / or presence of AAC6’Ib-cr

“+” indicates present; “-” indicates absent;
