# Supplementary material for: Overexpression of Efflux Pumps, Mutations in the Pumps’ Regulators, Chromosomal Mutations, and AAC(6′)-Ib-cr Are Associated With Fluoroquinolone Resistance in Diverse Sequence Types of Neonatal Septicaemic Acinetobacter baumannii: A 7-Year Single Center Study
Source: Front Microbiol. 2021 Mar 11;12:602724. doi: 10.3389/fmicb.2021.602724 (PMC7990795; doi:10.3389/fmicb.2021.602724)
Supplement: Supplementary file 3 [file Table_3.doc]

| **Strain No** | **Silent SNPs within AdeR not associated with overexpression of AdeABC efflux pump** | **Silent SNPs within AdeS not associated with overexpression of AdeABC efflux pump** |
| --- | --- | --- |
| A_112 | No silent SNPs were detected | V27I, V32I, A94V, L172P, F214L, S280A, Q281D, Q299R, Y303F, I331V, Q339K |
| A_113 | V120I | V27I, V32I, A94V, L172P, F214L, S281D, Q299R, Y303F, I331V Q339K |
| A_117 | Not tested | Not tested |
| A_124 | No silent SNPs were detected | V27I, V32I, A94V, L172P, F214L, S280A, Q281D, Q299R, Y303F |
| A_125 | V120I, A136V | L172P, N268H, Y303F |
| A_130 | Not tested | Not tested |
| A_131 | No silent SNPs were detected | V27I, V32I, A94V, L172P, F214L, S280A, Q281D, Q299R, Y303F, I331V |
| A_132 | Not tested | Not tested |
| A_136 | V120I | V27I, V32I, A94V, F214L, H 227 D, Q299R, Y303F, V331I, Q339K |
| A_138 | No silent SNPs were detected | V27I, V32I, A94V, L172P, F214L, S280A, Q281D, Q299R, Y303F, V331I, Q339K |
| A_145 | No silent SNPs were detected | L172P, D227H, , S280A, Q281D, Y303F, I331V , S341C |
| A_146 | A136V | V27I, V32I, L172P, F214L, S280A, Q281D, Q299R, Y303F |
| A_149 | V120I, A136V | V27I, V32I, A94V, L172P, F214L, H227D, S280A, Q281D, Q299R, Y303F, V331I, Q339K |
| A_150 | Not tested | Not tested |
| A_152 | Not tested | Not tested |
| A_153 | Not tested | Not tested |
| A_155 | No silent SNPs were detected | V32I, L172P, F214L, S280A, Q281D, Q299R, Y303F |
| A_157 | V120I | V27I, V32I, A94V, L172P,F214L, S280A, Q281D, Q299R, Y303F, V331I |
| A_158 | No silent SNPs were detected | V27I, V32I, A94V, L172P F214L, H227D, Q299R, V331I, Q339K, |
| A_159 | Not tested | Not tested |
| A_160 | Not tested | Not tested |
| A_161 | V120I | V27I, V32I, A94V, F188S, L172P, F214L, H227D, Q299R, Q339K, Y303F, V331I |
| A_162 | A136V | L172P, N268H, Y303F |
| A_163 | V120I, A136V | V27I, V32I, A94V, L172P, F214L, N268H, S280A, Q281D, Q299R, Y303F Q339K |
| A_166 | V120I, | L172P, F214L, Y303F, I331V |
| A_167 | V120I, A136V | L172P, Y303F |
| A_172 | V120I, A136V | The gene could not be detected |
| A_173 | V120I, A136V | L172P, N268H Y303F |
| A_177 | *adeR* could not be amplified | *adeS* could not be detected |
| A-179 | V120I, A136V | L172P, Y303F |

**Table S3.** Silent Single Nucleotide Polymorphisms (SNPs) in AdeRS regulator of AdeABC efflux pump in fluoroquinolone-resistant *Acinetobacter* *baumannii*

“Not tested”: Authors did not carry out the mutational analysis of AdeRS regulator for the isolates which either didn’t show overexpression of AdeABC efflux pump or *adeB* could not be amplified within them

The silent SNPs were polymorphisms which were also detected within the reference strains (*A. baumannii* AYE, ATCC 17978, ATCC 19606 and *A. baumannii* ACICU) and thus might not be associated with overexpression of the AdeABC pump.
